# A Review of IoT Firmware Vulnerabilities and Auditing Techniques

**DOI:** 10.3390/s24020708

**Published:** 2024-01-22

**Authors:** Taimur Bakhshi, Bogdan Ghita, Ievgeniia Kuzminykh

**Affiliations:** 1 Center for Information Management & Cyber Security, National University of Computer & Emerging Sciences, Lahore 54770, Pakistan; 2 School of Engineering, Computing and Mathematics, University of Plymouth, Plymouth PL4 8AA, UK; bogdan.ghita@plymouth.ac.uk; 3 Department of Informatics, King’s College London, London WC2R 2ND, UK; ievgeniia.kuzminykh@kcl.ac.uk

**Keywords:** Internet of Things, firmware auditing, reverse engineering, security testing

## Abstract

In recent years, the Internet of Things (IoT) paradigm has been widely applied across a variety of industrial and consumer areas to facilitate greater automation and increase productivity. Higher dependability on connected devices led to a growing range of cyber security threats targeting IoT-enabled platforms, specifically device firmware vulnerabilities, often overlooked during development and deployment. A comprehensive security strategy aiming to mitigate IoT firmware vulnerabilities would entail auditing the IoT device firmware environment, from software components, storage, and configuration, to delivery, maintenance, and updating, as well as understanding the efficacy of tools and techniques available for this purpose. To this effect, this paper reviews the state-of-the-art technology in IoT firmware vulnerability assessment from a holistic perspective. To help with the process, the IoT ecosystem is divided into eight categories: system properties, access controls, hardware and software re-use, network interfacing, image management, user awareness, regulatory compliance, and adversarial vectors. Following the review of individual areas, the paper further investigates the efficiency and scalability of auditing techniques for detecting firmware vulnerabilities. Beyond the technical aspects, state-of-the-art IoT firmware architectures and respective evaluation platforms are also reviewed according to their technical, regulatory, and standardization challenges. The discussion is accompanied also by a review of the existing auditing tools, the vulnerabilities addressed, the analysis method used, and their abilities to scale and detect unknown attacks. The review also proposes a taxonomy of vulnerabilities and maps them with their exploitation vectors and with the auditing tools that could help in identifying them. Given the current interest in analysis automation, the paper explores the feasibility and impact of evolving machine learning and blockchain applications in securing IoT firmware. The paper concludes with a summary of ongoing and future research challenges in IoT firmware to facilitate and support secure IoT development.

## 1. Introduction

Internet of Things (IoT) devices have become ubiquitous in a wide range of areas, including Industry 4.0, smart homes, smart cities, healthcare systems, the automotive sector, public services, and critical infrastructure [1,2,3,4,5,6,7,8,9]. The anticipated deployment of future generations of mobile access [10] and Low-Power Wide Area Networks (LPWAN) [11] technologies will see greater investment and drive the evolution of the IoT ecosystem [12,13]. Regardless of their popularity, the limited hardware and power capabilities of IoT devices lead to inherent challenges which affect security and device lifespan [14,15]. Many studies over the past decade focused on hardening IoT security at the network and application layers [16,17,18,19,20,21,22]; however, an important and often overlooked facet of secure IoT infrastructure is maintaining the integrity of IoT firmware.

A 2021 Microsoft review of the security landscape indicated that an increasing number of attacks focus on the IoT device firmware and BIOS (basic input/output system) due to a significant lapse and support for firmware security primitives [23,24]. Various categories of IoT vulnerabilities are directly linked to the firmware content and device capabilities. Firstly, due to their typical disposable nature, some devices cannot be updated or modified, which renders them vulnerable to issues discovered after their release. From the perspective of hardware capabilities, their fit-for-purpose design encompasses reduced storage and processing power, hence additional protection mechanisms may impede their functionality; further related to their design, their actual implementation cycle is not iterative and unlikely to be supportive of eliminating vulnerabilities identified after market release [24,25]. Due to all these inherent challenges, many traditional cybersecurity solutions cannot run on IoT hardware. Any vulnerable firmware present on IoT devices, coupled with their Internet-readiness, can therefore be exploited in a more streamlined and straightforward fashion; subsequently, any such device can be used as a bot, cause disruption, or be the starting point for other attacks. Some leading security companies, such as Checkpoint, offer products that investigate the security level provided by the firmware through runtime, weak credentials, and code checks against high-severity vulnerabilities from the Common Vulnerabilities and Exposures (CVE) list [26]. Potential attackers often consider alternative attack vectors, using domains and network endpoints that an IoT device firmware connects to, as infected firmware files might contain malicious payload as part of a more sophisticated attack. To summarize existing approaches to counteract these issues, our review outlines auditing methods that can be used to investigate the firmware of IoT devices against a wider spectrum of possible IoT firmware vulnerabilities.

Several prior studies and surveys have sought to ascertain and overcome security issues at the application and network layers of IoT systems [27,28]. While some of the studies including [25,29,30,31,32], focused on individual aspects of system architecture, emulation, operational and service security in IoT-ware, there is a fundamental requirement to comprehensively survey firmware security of IoT systems, to highlight existing challenges and discuss opportunities for future research. To this end, unlike previous studies, this paper provides a holistic view of existing IoT firmware deployments, prominent vulnerabilities, auditing techniques and limitations, and contemporary applications. Its focus on firmware also includes a comprehensive analysis of the different facets of firmware security to understand cross-domain concerns and aid future researchers and security practitioners. The primary contributions are listed as follows:

1. Deliver an overview of the related work in multiple areas of firmware security including reverse engineering, tool development, auditing mechanisms, and preliminary yet relevant work in machine learning. The paper couples the inherent limitations of IoT environments with existing tools and auditing mechanisms.
2. Present and analyze IoT firmware vulnerabilities across eight broad axes, their respective susceptibility triggers, and domain limitations based on prior literature. Although a number of prior studies do focus on particular aspects of the vulnerability spectrum, here the paper aims not only to define and categorize in terms of vulnerabilities, challenges, and corresponding mitigation measures, but also to map each of them with the exploitation vector and with the auditing tool that could help in identifying the vulnerability.
3. Undertake a detailed software vulnerability analysis, discussing reverse engineering methods and the latest solutions and frameworks available in the static and dynamic vulnerability analysis domain. Hybrid vulnerability auditing approaches are presented, along with the limitations of state-of-the-art auditing techniques and recommendations for improving scalability, coverage, support, and automation. This is an area that has been traditionally overlooked as past approaches delivered solutions aimed at open systems with no resource limitations, while existing reverse-engineering tools focused on eliciting system behavior rather than identifying vulnerabilities.
4. Summarize the state-of-the-art research in the area of IoT firmware security, including framework unification, multi-platform and multi-architecture support, tool management, machine learning and blockchain technology, all in the context of improving firmware security challenges, increasing vulnerability coverage, and providing potential recommendations for future research.

To deliver its contributions, the paper reviews the state-of-the-art efforts in securing IoT firmware, highlighting the causes behind its insecurity, along with a detailed discussion on the available techniques for security auditing and their efficacy. Compared to similar efforts, such as [24,30,31], the present review methodically discusses existing firmware problems, and investigates abstract vulnerability classifications that further motivate analyzing present assessment techniques and their limitations. The closest study related to our work is [32], which overviews firmware image re-hosting, emulation, and analysis. However, to the best of our knowledge, the present work is the first to comprehensively review and taxonomize the factors that contribute to or influence IoT firmware vulnerabilities, along with a discussion of existing static, dynamic and hybrid vulnerability auditing solutions, as well as the implications of future applications such as machine learning, deep learning, federated learning, blockchain technology, and framework unification.

The remainder of the paper is organized as follows. Section 2 provides a background overview on IoT firmware and related work. Section 3 details vulnerability influences in IoT firmware. Section 4 provides an overview of existing vulnerability analysis schemes and discusses the trends in auditing techniques. Section 5 explores the application of con-temporary technologies in securing IoT firmware, open research challenges, and provides recommendations for future research directions. The final conclusions are presented in Section 6.

## 2. Related Works

As mentioned in the introduction section, several studies catalogued IoT firmware security issues by aligning them with higher operational layers. This section selects prominent previous work in firmware security categorized according to primary focality in interface security, auditing methods, reverse engineering, emerging applications in blockchain and machine learning (ML), and commercial solutions. Table 1 summarizes a comparative analysis of the existing literature in the context of IoT firmware. The table also includes a further classification of the research scope as monolithic focusing on a single aspect, cross-sectional across multiple IoT operations, standardization efforts, or survey-oriented studies. Research and developments in each category are briefly described as follows.

- Interface security: Vulnerable interface identification in hardware, software, network, and application domains of IoT-ware represented the focality of studies in [29,31]. Some of the work in this area focuses on interface security and vulnerability solutions of consumer devices, detailing mechanisms for remote hijacking and control of IoT-ware, including surveillance nodes and general threats posed by IoT-specific malware [28,29,30,80]. Additionally, [31] provided a classification of existing solutions to detect IoT firmware threats, albeit without discussing corresponding solutions.
- Auditing techniques: Solutions describing the challenges in static [45,69,81,82] and dynamic auditing methods [33,38,83,84,85,86,87,88] have been proposed for IoT firmware vulnerability detection. Furthermore, to describing these fundamental vulnerability auditing techniques, some studies also highlighted the use of fuzzing technology and symbolic system execution to identify susceptibility in IoT-ware [84,89,90,91,92,93]. The primary efforts have been focused on assessing the effectiveness of different existing auditing methods and recommendations for developers/testers.
- Reverse engineering: Reverse engineering evaluation has been carried out on several commodity IoT devices to understand firmware vulnerabilities [94,95]. Employing fault injection, researchers have sought to identify the shortcomings of several vulnerabilities including weak authentication (password, PIN, etc.), device capability, and backdoors in IoT-ware [96,97]. System emulation schemes have also been the subject of research with a view to understand common challenges faced by developers and testers [38,87,98]. The tools and techniques employed for reverse engineering have been discussed in [19,25,94,95,96,99,100], providing basic discussion of pre-processing, de-compiling, unpacking, and evaluation techniques.
- Emerging applications: Ongoing advances in blockchain technology and machine learning technologies have also been topical areas of research in IoT-ware. Firmware data transmitted to IoT devices connected to a blockchain network is cryptographically proofed and signed by the true sender holding a unique public key, ensuring authentication and integrity of firmware [57,58,59,60,101,102]. When an IoT device needs to be updated, a smart contract [61] sends the hash or metadata file to that IoT device to obtain a copy of the update through peer-to-peer exchange with other nodes [58,59], or it is directly downloaded from the manufacturer’s server [62]. Bitcoin technology can also be employed to verify a firmware version before the update begins and to acknowledge a transaction before the IoT device can download and install it [57,101]. The studies [63,64] proposed direct and indirect firmware update distribution based on Ethereum blockchain. Similarly, Skipchain blockchain technology has also been proposed for secure trusted firmware updates using smart contracts [103].

Firmware identification is vital in preventing spoofed firmware packages. Machine learning algorithms are used for identification and classification of IoT image fingerprinting [70], according to vendor or device type [71]. Greater ML-based automation significantly reduces the latency involved in reverse engineering maneuvers such as firmware decompression [72,104].

- Commercial developments: In the commercial realm, TrustZone [73] by ARM has provided users a hardware-based security extension establishing a root of trust (RoT) and cryptographic services to securely store critical (firmware) data, which is an improvement over conventional trusted platform modules (TPM). TrustZone allows a wider set of hosted sensitive services driven by (hardware-based) isolation; however, an ever-expanding set of threats from secure-mode operation is not uncommon [74]. Similarly, Intel Turstlite [75], a generic security architecture suited to low-power embedded devices, allows remote management, authentication, and over the air (OTA) updating as well as remote attestation [76]. Among low-cost solutions, IoT-ware memory access control can also be implemented using SMART [79], using a ROM measurement routine with a secret key to provide remote attestation. However, SMART does not specifically deal with memory access violations or provide provisions for updating the attestation code [77] and, as discussed in [42,78], the verifier can also be malicious while the prover is benign, a significant limitation of remote attestation.

Compared to earlier studies surveying specific aspects of IoT-firmware, to the best of our knowledge, the present work is the first to provide a comprehensive survey of IoT firmware, holistically treating auditing techniques and tools in secure firmware management and delivery. The subsequent sections describe the individual aspects of the above highlighted research streams.

## 3. Firmware Vulnerability: Influences and Challenges

**Figure 1:**
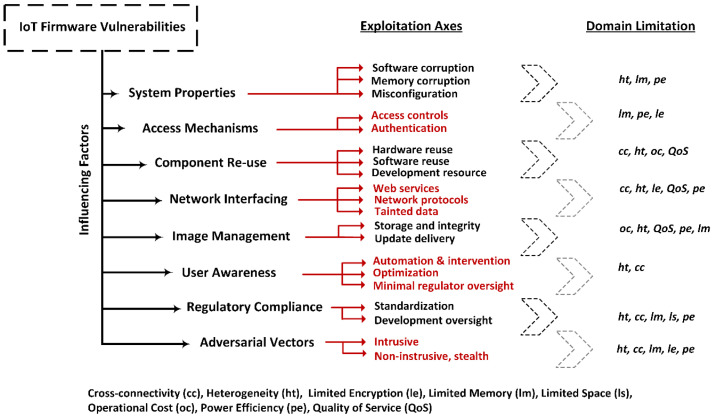
IoT firmware vulnerabilities—influencing factors.

The amalgamation of multiple technologies embedded in IoT lends greater susceptibility of IoT devices to several attack vectors. This section discusses IoT-ware vulnerabilities from a system and operational perspective based on the primary influencing factors in the existing literature [29,31,100]. Prior studies had various approaches for clustering vulnerabilities based on their preferred discriminator: the attack source (physical, local, network, Internet), the nature of the threat (hardware, operating system, software, interaction), the TCP/IP layer, the environment (off-the-shelf, corporate), or the impact (denial of service, bot harvesting, impairing QoS, data leakage). Our approach focuses on the IoT ecosystem, considering the design, development, and management of IoT devices, crossed with the access and operational characteristics of such an ecosystem. The design and development encompass the hardware, operating system, software, communication, and configuration issues, with the management adding in the additional systems required for normal lifetime functionality. The other direction focuses on user interaction component and brings in the legal framework, access, and any adjacent environments. Based on this, we classified the influencing factors in eight broad categories namely, system properties, access controls, hardware and software re-use, network interfacing, image management, user awareness, regulatory compliance, and adversarial vectors illustrated in Figure 1.

Drawn from prior studies, the prevalent discriminator for vulnerabilities is the attack vector they trigger; therefore we built the exploitation triggers into our classification, which are referred to as Exploitation Axes. At the other end, each vulnerability is driven by a domain characteristics and limitations; this is essential within the scope of our study because it emphasizes the differences brought in by an IoT ecosystem versus a traditional IT environment. Guided by the headings of the vulnerability areas, the following sections will discuss the encompassing exploitation axes and domain limitations.

### 3.1. System Properties

System software exploitation remains one of the fundamental avenues to target and exploit IoT firmware vulnerabilities.

- Software corruption: IoT firmware is inherently susceptible to software corruption, such as coding bugs introduced at service initiation or operation or during upgrades [29]. Coding bugs may introduce pointer violations, and type/format confusion, while programming related issues can also lead to malicious code injections, running of privileged commands and system failures. Tainted data and unexpected input can alter device behavior and further expose it to firmware threats.
- Memory management: Inefficient or corrupt coding can also lead to integer and buffer overflow, a common cause of security vulnerabilities further exacerbated by memory constraints inherent in IoT-ware [105]. Application requirements also may dictate implementing safety critical services in separate hardware chips [93,106]. While hardware-based trust management (HTM) is considered an optimal solution, the spatial and financial cost again might render it unfeasible for IoT-ware. Adopting HTM is also limited by the typical absence of dedicated Memory Management Units (MMU) in IoT systems, leading to frequent memory violations. Service isolation can also be offered solely in software, utilizing virtual memory and enabling monitoring of device sub-systems, allowing wider cryptographic support despite code-sharing on a single processor [79,106]. Additionally, dynamically establishing a root of trust by modifying the existing microcontroller units (MCU) using a hardware-software co-design approach is being used to allow greater flexibility and lesser spatial, as well as memory consumption for secure code execution. Using remote attestation techniques [76,78,107], detection and disabling of malicious code can be actioned before compromised execution.
- Misconfiguration: Domain limitations including limited memory, power efficiency and device heterogeneity need to be recognized during system configuration to mitigate some of the system vulnerability exploits discussed earlier. Misconfiguration of the system may lead to a successful exploitation.

### 3.2. Access Mechanisms

Access, authentication, and credential management all play an essential role in patching IoT nodes, as devices can be located in remote environments where manual (local console) updates are economically infeasible, requiring over-the-air-update mechanisms.

- Access control: IoT firmware access requires well-defined policies and suitable encryption to mitigate against password, certificate, or encryption key threats. The device manifest, containing author information and firmware update policy, if left un-encrypted, can lead to accessing, altering, or deleting vital metadata required for future authentication and upgrades to device firmware. Similarly, public certificate servers utilizing SSL (Secure Sockets Layer) certificates for provision of IoT-ware security may lead to man-in-the-middle attacks if repeatedly reused for a range of devices [81]. While vendors may also incorporate backdoor channels or push mechanisms to access devices for regular updates, such channels, if not protected by adequate credential management, may result in compromising device firmware or device data [85,108].
- Authentication: IoT-ware attacks due to weak authentication mechanisms are rather common [31]. Misconfigured and erroneous authentication routes allow control and jeopardizing of normal operation [108]. Weak authentication is usually due to resource constraints, allowing limited authentication schemes to conserve memory and processing power.

### 3.3. Component Re-Use

- Hardware and Software Re-use: Hardware and software components re-use, including off-the-shelf boards, circuitry, sensors, bootloaders, or software libraries, is prevalent among vendors to reduce development time and associated costs in the IoT domain [88], while inadvertently overlooking vulnerabilities arising due to heterogeneous cross-connectivity. In multi-controller systems, firmware from different manufacturers requires comprehensive security analysis and testing of each individual component. Firmware vulnerabilities in one controller or in exploitable software can lead to cascaded threats disrupting the entire operation and to the mass production of a range of insecure IoT devices [18].
- Development Resource: An ever-evolving set of IoT applications has also generally led to vendors frequently employing developers with limited expertise in developing high-quality firmware [16,34]. In addition, vendors also tend to overlook firmware vulnerabilities in favor of overall device usability and performance.

### 3.4. Network Interfacing

IoT devices interact with other heterogeneous systems over several interface types and networking protocols. However, this may translate into application programming interfaces (APIs) and protocol susceptibilities, presenting potential attackers with opportunities to compromise device functionality as well as accessibility. In order to understand the impact at each level, it is worth observing how each communication level may introduce its own attack opportunities.

- Web Services: IoT devices communicate with cloud, fog, edge computing and monitoring systems over a range of web APIs. Insecure, poorly designed web services remain one of the leading causes of device exploitation, allowing service interruption via application-level and firmware-based attacks [33,35,100]. Prominent malware such as IoTReaper have successfully exploited IoT web interfacing to launch wide variety of attacks on device-ware [109]. Limited resources again hamper the adoption of multi-factor authentication incorporation in IoT-web interactions [46].
- Network Protocols: Vendors use a wide range of standardized and proprietary network protocols that, when combined with reusable hardware and software components, may lead to propagation of existing security issues in IoT-ware. Poor management of device network configuration, such as leaving open unused ports, may lead to security issues. The security firm Kaspersky reported that, in the first half of 2023, honeypots recorded that nearly 98% of the network-related attacks on IoT-ware occurred on the unsecure Telnet interface [43]. Over-the-air updates need to employ standardized and tested protocols that offer greater protection against man-in-the-middle and spoofing attacks while patching firmware.
- Tainted Data: The sensor and actuation services process incoming data that may require acquisition, perusal, validation, processing, and sanitization through associated fog and cloud nodes. Data acquired from sensory or actuator portals, if tainted or malformed, can overwhelm device operability and expose the device firmware to security risks [110].

### 3.5. Image Management

Firmware image management is vital for the longevity and secure operation of IoT-ware. Inefficient non-redundant firmware storage and upgradation schedules coupled with suboptimal configuration parameters, will negatively influence IoT firmware integrity.

- Storage Integrity: IoT device firmware requires image storage integrity as well as secure distribution and updating to mitigate the threat exposure. Despite improvements in OTA mechanisms, device developers are generally reluctant to provide security patching as a continual maintenance service [47]. Given the significant lifespan of IoT operations, devices may be running obsolete firmware several years old that has several discovered, widely acknowledged flaws. Where OTA updates and encryption mechanisms are available, the network protocols also need to be tested for security compliance and suitable encryption. As an example, investigations by the security community identified that the update protocol for the popular FitBit devices is prone to hacking despite using end-to-end encryption [111]. Firmware image integrity is a strict requirement to avoid attempts at flashing or modifying existing images from unwarranted sources, protecting image confidentiality from adversaries recovering plain text binaries.
- Update Delivery: The process of firmware update, where available, can be used as an attack delivery option, as it can be initiated by the customer, pushed from a server, or follow a hybrid approach; in addition, vendors may introduce provisions for backdoor updating of device firmware [64,103,112]. While not an intrinsic vulnerability, having firmware downloads available publicly may also offer an insight into the libraries, settings, and functionality to launch sophisticated attacks. Lack of coordination between the operating parties, server and network downtime, and device outages can also lead to inconsistencies in update tracking, causing unnecessary delays to firmware updating.

### 3.6. User Awareness

Firmware updates frequently involve complex decision-making processes, such as the re-certification of tested code, and once the device has been deployed, consumer consent in upgrading to any of the new functionalities.

- Automation and Intervention: An efficient device update process requires a balance between automation and human intervention, whereby large-scale updates should be performed using minimal manual intervention. To optimize decision making, necessary provisions for manual intervention can be kept, while maximizing de-facto upgrade policies using dynamic updates to be applied as released. Users can also be incentivized to update by flagging the risks that they expose themselves to in case of non-compliance.
- Optimization: Incorrect operational settings such as disabling or reducing event logging to conserve energy makes post-incident analysis difficult and prone to errors. A significant number of IoT vendors provide devices without any user guidelines for updating configuration parameters based on usage. Opting for default settings, ranging from generic authentication passwords, switched off update notifications, or outdated web applications, vendors pass the responsibility and burden of device firmware updates to the end-user. However, as widely acknowledged in the literature, firmware adjustments are rarely considered or applied by everyday users [23,28,112]. A general improvement in the set of guidelines to provide the user with sufficient information to secure their devices is nonetheless vital and consortiums such as IoT Alliance Australia issued specific user guidelines on the maintenance and operation of IoT firmware updating and help in identifying firmware hijacking [113].

### 3.7. Regulatory Compliance

The IoT paradigm is still an emerging technology subject to ongoing standardization. This section introduces some of the existing efforts in standardization and compliance.

- Standardization: Existing IoT-ware regulations have been introduced by commercial and governmental organizations including OWASP (Open Web Application Security Project), IoT Security Foundation, and NIST (National Institute of Standards and Technology). Standardization bodies have provided operational guidelines as well as best-practice mechanisms to provide secure IoT systems; however, these have not been widely adopted due to limited regulation. On a similar note, inadequate and inefficient compliance resulted in insecure booting, minimal or no encryption, and outdated firmware. Enforcing security compliance as part of IoT-related products engineering, development frameworks, and business policies requires greater regulatory oversight by governmental and non-governmental bodies which are usually beyond the scope of standardization organizations.
- Development Oversight: Vendors with inadequate experience in the IoT domain have been mass producing devices without adequate security inclusion [94,114,115]. A separate category of oversight challenges is linked to the design and manufacturing process. Hardware device manufacturing and software provision tend to be rather independent processes and coordination issues between original device manufacturers (ODM) and original equipment manufacturers (OEM) may result in overlooking firmware flaws. Code developed and supplied by ODMs may contain security loopholes that, when used and implemented by OEMs, may result in replication across thousands of commercial devices [16,35].

### 3.8. Adversarial Vector

It is important to consider adversarial models when documenting the vulnerability triggers of IoT-ware.

- Local and Remote Vectors: Remote or over the network adversarial factors can infect systems via malware, while local adversaries can eavesdrop and interfere with device communication [107]. Stealth-based adversaries can attack either from closer physical proximity or remotely, masquerading as an authentic entity and gain unwarranted access to the IoT ecosystem [79].
- Side-channeling: Similarly, side-channel attacks can be carried out by a physical non-intrusive entity, while an intrusive adversary can completely overtake an authentication mechanism to prove its identity to an IoT device aiming to solicit information or exploit device behavior through hardware-software modification [79].
- Hybrid Designs: Dedicated hardware and software security have associated cost implications; the inherent spatial, financial, and power efficiency compromises for IoT-ware require careful trading off. A combinatorial approach using a mix of hardware and software-based controls to address adversarial threats is often considered to be a more viable option compared to purely hardware-based security or an entirely software-oriented security primitive [107].

The above discussion provides a non-exhaustive list of the major vulnerability influencing factors, ranging from system and network properties to firmware image management and user-awareness concerns. In the following section we specifically consider the state-of-the-art firmware vulnerability auditing tools and technologies.

### 3.9. Domain Limitations and Associated Impact

As mentioned, IoT devices are inherently limited devices, but these limitations span across multiple areas, as highlighted in Figure 1. From the hardware perspective, they have limited memory (lm) and limited storage space (ls) due to their reduced manufacturing cost; a significant direct impact of these characteristics is that such devices cannot typically employ additional security monitoring processes. Also under this heading are their limited encryption (le) capabilities, which directly impact protection mechanisms thar are computationally intensive or require specialized hardware. Given their wide deployment, each IoT device must also benefit from a low operational cost (oc) and must deliver excellent power efficiency (pe), both components also having a direct impact on any security mechanisms that users may wish to deploy but, in addition, also severely impacting any support, update, or monitoring infrastructure or associated management costs aiming to keep them up to date. The limited hardware also impacts their ability to perform any additional security and update functions beyond their primary purpose, which has specific quality of service (QoS) associated constraints. All above listed points relate to individual devices; expanding to the overall IoT ecosystem, there is a wide range of devices from a variety of manufacturers, which leads to significant heterogeneity (ht) and cross-connectivity (cc) to allow them to operate. While beneficial from a market competitiveness perspective, these two constraints have a direct impact on harmonizing defense mechanisms and they also make regulatory compliance virtually impossible to achieve.

## 4. Vulnerability Auditing

Firmware auditing is a manually intensive task, requiring assessor expertise in reverse engineering (RE) and a multitude of static (SA) and dynamic analysis (DA) techniques [110]. Prior to vulnerability analysis, the respective firmware needs to be systematically processed to ensure its compatibility with the chosen auditing method. Once processed or re-hosted, the firmware is subjected to vulnerability auditing testing processes for accurate determination of inherent weaknesses. The completeness and accuracy of vulnerability auditing is subject to several associated challenges in reverse engineering tasks, as well as the adequacy of state-of-the-art vulnerability analysis mechanisms. The present section overviews the generic firmware reverse engineering process, discussing existing analysis techniques and their respective limitations.

### 4.1. Reverse Engineering

Firmware source code is usually not readily available for vulnerability auditing. As part of the firmware examination process, the first and foremost step is to perform a series of reverse engineering tasks involving binary file acquisition, unpacking, and de-compilation to access the source code [16,65,100,108]. Table 2 provides an overview of existing tools involved in reverse engineering process along with their performance caveats.

#### 4.1.1. Firmware Acquisition

Firmware can be acquired from a vendor repository, locally extracted from a device [94], or intercepted and saved during OTA updating [19]. Firmware acquisition automation using web-crawling and scripting techniques is also possible [81,108], although dedicated FTP-based image servers remain the preferred option [83].

Code can also be acquired from devices through JTAG and UART ports or by using forensic analysis techniques [85,94,127]. Device manifests and update servers may schedule regular upgrades of device firmware using OTA updates [19]. Depending on encryption, firmware data (update) transfer mechanisms can allow vulnerability analyzers to record and store data during the update process through packet sniffing or mirroring [128]. Establishing central repositories that aggregate firmware code from multiple vendors to expedite and scale auditing procedures remains a long-standing tester requirement [114].

#### 4.1.2. Firmware Unpacking

The criteria and scheme for binary packing is usually vendor-specific and considered proprietary [33,81,108,129]. Some of the common challenges faced by testers during unpacking include file encryption, obfuscation [44], compression using non-standard schemes, or a monolithic multi-feature systems containing kernel, OS and IoT applications bundled together [33]. Each of the unpacking concerns require independent selection and application of tools, the foremost being entropy analysis to determine the encryption or obfuscation techniques. The overall confidence in the output generated is, however, minimal, requiring repeated analysis by domain experts for unpacking [71]. Some of the other prominent tools used for unpacking (summarized in Table 2) include Binwalk [116] and BANG [117] using recursive unpacking, while FMK [118] and FACT [119] focus on Linux-based platforms. Multi-faceted tools such as ANGR [120] can also be utilized as part of reverse engineering and analysis processes. ANGR is a python-based platform offering binary analysis, automated firmware unpacking, control flow analysis, symbolic execution, and compatibility with Linux, Windows, and MAC platforms. The operational capability of ANGR is only limited by either OS-specific or inconsistent backend support. Successful firmware acquisition and unpacking is followed by source code generation.

#### 4.1.3. Decompiling

Decompiling machine code is needed for greater human readability in a higher-level language and comprises disassembly, data flow, control flow analysis and data type inspection [130,131].

Machine code is first converted to a low-level assembly equivalent. Modern compilers are capable of separating executables from data; however, if data are placed in the executable section, it may result in inefficient execution code and data isolation.

After disassembly, during lifting and data flow assembly processing, the code is translated to a higher level internal representation. Control flow analysis can also employ control flow graphs, allowing data type identification in the code. Debugging is sometimes also used to analyze sections of particular security interest [39]. Popular de-compilation tools include Radare2 [122] and Binary Ninja [121] and provide binary analysis capabilities with (optional) GUI support. IDA Pro [124] and Ghidra [123] have multiple features including interactive disassembly and multi-architecture support. KLEE [48] uses symbolic VM processing (LLVM) compiler with relatively heavy resource consumption.

#### 4.1.4. Challenges

The impact of the issues relating to the acquisition, unpacking and de-compilation process is amplified by a number of additional challenges highlighted as follows.

- Packing logic: packers do not modify the code functionality, making presentation of the code sequential and not readily human-legible for security analysis. Therefore, use of automated dynamic analysis as opposed to manual perusal can yield better results, providing auditing scalability for a multitude of firmware solutions [104]. Testing frameworks, including FAT [49] and QEMU [125], simplify the analysis by incorporating several vulnerability assessments tools and emulation.
- Mitigation techniques: In addition to cryptic packing, vendors may resort to de-compilation mitigation, adding to firmware source inspection obstacles.
- Metadata unavailability: Masquerading device meta-data to avoid hardware-based hacking can inadvertently complicate the security auditing process [94,129] by limiting information on product release, update log and version number, and hardware architecture for de-compiler selection [132]. Intuitively assuming protocols, OS and libraries and other data inputs are used to analyze the device for security vulnerabilities is therefore common, as is brute-force fuzzing using genetic algorithms such as the American Fuzzy Lop (AFL) fuzzer [126] that aids when randomizing input testing. The scope, applicability, and operational capability of auditing techniques remains vital to firmware vulnerability assessment and device protection.

### 4.2. Auditing Techniques

Auditing techniques encompass methodologies for vulnerability analysis of IoT firmware. In the existing literature [32,33,66,81,83,85], auditing techniques can be broadly divided into static and dynamic auditing schemes. Table 3 presents a comparative analysis of the schemes against the auditing features.

#### 4.2.1. Static Analysis

Static analysis involves manual and intensive scanning of the source code against ruleset patterns to identify coding errors [36,66,133]. Static analysis, therefore, does not involve the actual execution or emulation of firmware and does not require the auditor to have physical access to IoT devices for scrutiny [44]. Typical vulnerabilities determined using static analysis include invalid references, buffer overflows and memory corruption flaws [67], segmentation faults, and uninitialized variables [68]. To reduce cost and time, auditors can use tools to automate sub-processes, sometimes at the risk of greater false positives. Similarly, code obfuscation and encryption techniques employed by device manufacturers can impede static vulnerability analysis [134]. We analyze existing static analysis strategies and tools over the past decade that we summarized in Table 4.

Historically, we can divide existing static analysis strategies into six categories: Manual analytics, Automation and parallelism, Parsing-based analysis, Control flow graphs, Machine learning approaches, Determining backdoors.

A typical example of a manual analysis tool is woodpecker, introduced in 2012 for Android applications [135]. Although the tool itself was not intended to find firmware vulnerabilities, it did find permission leaks in pre-loaded applications. Later, in 2014, the work performed by Costin et al. [81] laid the basis for firmware vulnerability detection, including an extensive study of more than 32,000 firmware images. After statically analyzing the images, the authors were able to detect over 693 different vulnerabilities, including 38 zero-day vulnerabilities.

Firmalice, another binary analysis tool proposed in [108], used an automation and parallelism approach that slices a program and uses a symbolic execution engine to execute parallel functions for recording vulnerabilities. The tool has the ability to understand security policies as well as identify privileged instructions.

A parsing-based analysis group was introduced by Parser Identification in Embedded Systems (PIE) [69], which is a tool for detecting functions while parsing components and complex code. Before any classification can be performed on the parsed components, the firmware binary code is converted to an intermediate language via LLVM, thereby allowing PIE to analyze the firmware of embedded systems without any documentation or source code. PIE can be used for detecting exploitable bugs, extracting protocol specifications, and finding hidden commands, and has been widely tested on user devices such as GPS systems, power meters, hard disks, and PLCs (Programmable Logic Controllers).

As a follow up to Firmalice, Shoshitaishvili et al. proposed ANGR [120], enabling both static and dynamic analysis as briefly described earlier on; ANGR remains popular among many other tool frameworks for carrying out firmware analysis using binary control flow graphs (CFG). Following a different approach, FirmUp [82] performs static vulnerability analysis of firmware images using CFGs and, additionally, firmware slicing to find the exact location of vulnerable procedures. Using reverse engineering tools including Binwalk, IDA Pro, and ANGR, researchers claimed to have outperformed other static analysis methods by an average margin of 45%. CFG schemes allow auditors to systematically inspect firmware; however, scalability remains a concern with an ever-increasing diversity in firmware.

Machine learning has been used to enable greater automation by incorporating pattern recognition in existing static analysis techniques. In 2016, Feng [136] introduced an algorithm called Genius to solve the scalability problem with control flow graphs using a combination of machine learning and computer vision techniques. In 2017 Xu et al. [40] developed a neural network-based approach, named Gemini, seeking to outperform algorithms such as Genius [136] using a proof-of-concept implementation. The aim was to reduce the classifier training time while finding a significantly higher number of vulnerabilities in firmware images. In 2019, Wang et al. proposed a two-stages firmware vulnerability detection based on code similarity [41] but the study did not categorically prove greater accuracy compared to Gemini.

A set of static analysis tools have also been developed to determine undocumented functionalities hidden in firmware. A prominent example is HumIDIfy [45], which uses ML to identify any hidden functionality, using a set of profiles with expected firmware behavior and a binary functionality description language that compares these with the real-time code behavior. If variations are found between expected and real-time behavior, then the firmware is assumed to have hidden functionality. Although it is a novel approach, it cannot be regarded as a complete solution because it requires expert human knowledge and firmware metadata to avoid generating a substantial number of false positives.

Another common vulnerability in firmware development is the use of backdoors. Stringer [134], a tool based on automatic static analysis of firmware, seeks to address this problem. In a similar study, a tool named Universal firmware vulnerability observer (UFO) [137] was proposed and could be used for firmware vulnerability, reversing, determining password leakages, and finding backdoors using a newly developed algorithm called Shell Script Dependency (ShDep). UFO can be used to ensure that embedded IoT devices follow the IoT specific security and privacy standards such as OWASP, UL-2900 [138], and ICSA Labs [139]. During UFO validation, 96% of 237 devices considered were successfully reverse engineered and more than 70 were found to have common vulnerabilities. Although UFO cannot reverse engineer obfuscated or encrypted firmware, it claims to have better firmware file system extraction when compared to existing tools.

#### 4.2.2. Dynamic Analysis

The dynamic schemes execute firmware code allowing auditors to observe system behavior without requiring access to the program internals information. Dynamic analysis requires metadata information to optimize firmware emulation. However, images cannot always be emulated without knowledge of the underlying architecture, therefore dynamic analysis does not scale well when automated emulation is not possible, as it would require repeated customization of emulation and configuration setup. Typically, dynamic analysis is employed when source code is unavailable or de-compilation is unsuccessful. We will analyze existing the prominent technologies and tools used for dynamic analysis listed earlier in Table 5. There are several well-used methods for conducting the dynamic analysis: peripheral emulation, symbolic execution, abstraction modelling and fuzzing techniques.

FIE [92] was developed to scrutinize memory locations of peripherals using invocation of interrupt handlers to observe behavior. FIE was built using KLEE symbolic execution engine [48] and is micro-controller specific. FIE keeps records of all previously analyzed states, filtered using state pruning and memory smudging. State pruning helps remove redundant state executions for even small firmware images, while memory smudging allows FIE to recognize loop counters and replace them with symbolic variables to help with greater code coverage.

Symbolic execution is a rather slow yet powerful technique to determine equations capable of defining as well as fully describing the stagnant and operational state of firmware in real-time. Using symbolic execution, peripherals are emulated, and input is generated for execution and testing in real-time. Tools such as Laelaps [98], µEmu [140], or Gerbil [88] can run various embedded device software without coding any specific device related information into the emulator. Unknown peripheral registers are considered as symbols and the input firmware image is symbolically executed to infer rules responding to unknown peripheral access types. The rules are further stored in a database that can be referred to during firmware analysis. The Gerbil [88] is an extension of the ANGR [120] static tool and was used to test privilege separation vulnerabilities in everyday smart devices.

**Table 5:** Auditing strategies and tools for dynamic analysis.

Tool	Year	Analysis Method	Target Vulnerab.	SC.	UV.	PV.	Architecture
FIE [92]	2013	Symbolic execution	Memory bugs	Partly	Yes	Yes	MSP430
Avatar [85]	2014	Emulation	Any	Partly	Yes	Yes	Multiple
Firmadyne [141]	2016	Emulation	Multi-domain	Yes	Yes	Yes	ARM MIPS
Dynamic auto. [33]	2016	Emulation	Web vulnerability	Yes	Partly	Partly	Multiple
Multi-stager [99]	2016	Binary analysis, virtualization	Industrial IoT systems	Yes	Yes	Yes	Multiple
FIoT [142]	2016	Symbolic execution	Memory corruption	Yes	Partly	No	Multiple
P2IM [86]	2017	Abstraction model	Any	Partly	Yes	Yes	Multiple
DICE [38]	2021	Abstraction model	Any	Yes	Yes	Yes	Multiple
HALucinator [87]	2020	HAL, Emulation	Any	Yes	Yes	Yes	Multiple
PRETENDER [88]	2018	Emulation	Any	Yes	Yes	Partly	Multiple
Laelaps [98]	2020	Symbolic execution	Any	Yes	Yes	Yes	Multiple
µEmu [140]	2021	Symbolic execution	Any	Yes	Yes	Partly	Multiple
Gerbil [88]	2019	Symbolic execution	Any	Partly	Yes	Yes	Multiple

SC: Scalability issue, UV: Unknown Vulnerability Detection, PV: Platform Versatility.

Several multi-utility frameworks were developed to execute the dynamic analysis supported by full system emulation via QEMU [125], which emulates the I/O and kernel operations. One such framework is Avatar [85], able to perform dynamic analysis of embedded device firmware and having equal applicability in the IoT domain; however, it requires real hardware to discover vulnerabilities slowing execution of the entire procedure, which is adding to its scalability concerns. In contrast, the framework proposed by Costin et al. [33] can identify vulnerabilities in Linux-based systems without requiring actual hardware by testing the embedded web interfaces with readily available open-source security scanner tools such as Zed Attack Proxy (ZAP), Nmap, and Nessus, followed by Metasploit for exploiting vulnerabilities. Firmadyne [141] focuses on Linux-based firmware vulnerabilities; it can crawl vendor websites searching for firmware images along with their metadata using manually written scripts. After downloading the images, it extracts the kernels and performs dynamic analysis methods to find and exploit vulnerabilities on the emulated firmware.

Expanding to industrial IoT-ware, the dynamic framework proposed by Palavicini et al. [99] uses a combination of methods, including binary analysis tools, cyber reasoning system, fuzzer, as well as security analysis virtualization solutions such as OpenPLC, Firmadyne, and QEMU. The study proposes a three-stage approach, starting with the extraction of the firmware blob to extract code for emulation, further emulating the code, and analyze the results for vulnerabilities using a number of techniques such as fuzzing and symbolic execution. This analysis results in finding backdoors, information leakage and code for creating botnets. A similar multi-stage approach is used in FIoT [142] and it allows the identification of memory corruption issues in constrained IoT firmware.

Feng et al. [86] proposed the Processor-Peripheral Interface Modeling (P2IM) software framework based on an off-the-shelf fuzzer channeling input to firmware binary for auditing. P2IM uses abstraction modelling of peripheral devices to generate firmware models; it also employs information from manufacturer device documentation to understand acceptable processor-peripheral interface inputs. An extension to P2IM, the DICE framework [38] is used for emulation of direct memory access (DMA) channels in firmware analysis. The framework is hardware-independent, identifying and abstracting DMA input channels as firmware communicates with source and designation DMA transfer points in the DMA controller. DICE can manipulate the input transferred via DMA for analysis and is integrated in the P2IM framework.

Abstraction modelling is also used by HALucinator proposed by Clements et al. [87]. HALucinator uses high-level replacements of the hardware abstraction layer function and locates all the library functions using binary analysis of a firmware and library matching techniques to infer functions. HALucinator was validated using American Fuzzy Lop fuzzer, employing genetic algorithms for greater use-case coverage; during the validation experiments, it reported multiple previously unknown firmware library vulnerabilities.

While most methods promise multiplatform and multi-architecture analysis, real-test cases and reported results have mostly focused on limited classes of firmware. To fully appreciate their readiness, a comparative analysis of alternate approaches and tools requires the same firmware as well as a wider set of architectures, characteristics that are unfortunately absent in existing studies.

#### 4.2.3. Hybrid Proposals

Hybrid approaches, amalgamating static binary analysis with dynamic real-time investigations are a valuable option for greater auditing coverage. A hybrid combination of auditing techniques can be used to increase unknown vulnerability detection efficacy. From a practical perspective, multiple systems can be considered hybrid; Costin and Zaddach’s work [28], described earlier, is a combination of dynamic and static analysis that aims to achieve full automation. Similarly, DroidRay [50] was developed to discover malicious code in Android devices by relying on dynamic analysis during APK files checks and on static analysis during scanning for viruses. Shoshitaishvili et al. [143] implemented Mechanical Phish, a hybrid vulnerability detection framework that combines fuzzing with symbolic execution to find bugs while satisfying specific and general checks required by the tested programs.

Hybrid techniques can also rely on fuzzing, using malicious input patterns to trigger unexpected device operation, essentially stress-testing system security. IoTFuzzer [129] is one such framework that uses a black-box approach to detect possible memory corruption vulnerabilities. It sends probing messages to the IoT device and, when crashing, collects the generated error messages. Zheng et al. also implemented a grey-box fuzzer called Firm-AFL [51] that supports firmware sets that can be emulated through Firmadyne [141] and cannot be fuzzed via Firm-AFL. The authors in [84] developed a vulnerability-oriented fuzzing tool named FIRMCORN which uses a vulnerable-code search algorithm to find vulnerabilities in IoT firmware. Despite these recent developments, inherent scalability issues incorporated in hybrid dynamic and static analysis will continue to be a concern for fixed input as well as fuzzing-based techniques.

### 4.3. Discussion of Auditing Techniques

Dynamic analysis is preferred by practitioners over static analysis, despite the inherently high vulnerability determination efficiency of the later, because complex reverse engineering and tight software–hardware coupling raise additional challenges for static auditing [38]. Over the recent years, fuzzing and hybrid methods have also gained wider adoption. Frameworks incorporating static, dynamic and hybrid techniques can be developed for accurate identification over most of the vulnerability axes listed in Figure 1. One important facet to consider while amalgamating different solutions is the availability of utilities for a specific underlying platform. For the majority of the solutions discussed in previous sections, Linux platforms remain prevalent while architecturally most solutions support ARM and MIPS with partial support for others. ARM, MIPS, and x86 are architectures with different instruction sets used in the design of computer processors.

In relation to the scope of the analysis, as discussed in Section 3, there are several classes of vulnerabilities that need to be audited and analyzed, including system properties, access mechanisms and networking, code reusage, and user awareness. While some vulnerability triggers such as authentication bypassing, hard-coded credentials, and memory corruption have been the subject of interest due to their ubiquity, less frequent alternatives are often overlooked during auditing. Misconfiguration, user-awareness, lack of regulatory compliance and standardization, as well as tainted data input and essential image management have received lesser attention due to the complexity of a potential investigation and variability across the spectrum of IoT-ware in use. Table 6 provides a summary of the eight different vulnerability classes along with their prominent auditing primitives, respective platform, and architecture support.

Due to heterogeneity of IoT-ware, auditing tools vastly focus on identifying and replicating recognized attacks, while only a few solutions focus on finding zero-day vulnerabilities. The versatility of existing tools is questionable, as most of them only cover a particular class or subclass of vulnerabilities and may not be easily extended to cover others. In hindsight, some solutions such as ANGR [120], Genius [136], Gemini [40], Avatar [85], and DICE [38] can detect numerous vulnerabilities due to their underlying methodology, but may also exhibit a high rate of false positives unless used by domain experts. Several solutions discussed earlier employ a wide range of methods for firmware analysis, including function profiling, program slicing, inter-relating shell scripts, code snippet emulation, and augmented process emulation. While beneficial for individual tools, establishing and developing a similar critical mass of equivalent human expertise in such a wide variety of techniques is very challenging. The complexity involved and the associated human expertise required can directly impact auditing results. In terms of future trends, research is increasingly focusing on machine learning and blockchain technology. ML and blockchain can, to an extent, bring further flexibility, adaptability, and automation to firmware auditing. However, harnessing the full spectrum of potential applications of these technologies remains an open initiative.

## 5. Contemporary Research and Open Challenges

**Figure 2:**
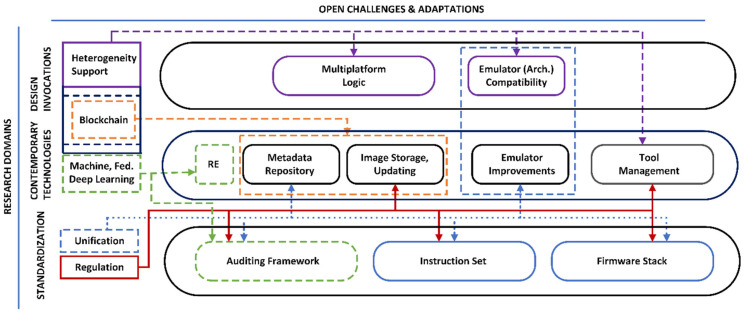
Future research avenues, open challenges, and intersecting themes.

This section examines firmware vulnerability challenges, as identified in the previous sections. The relationship between state-of-the-art and contemporary research is streamlined across three integral components: standardization, technology redressal, and design innovations. Figure 2 illustrates future research directions and corresponding challenges. The arrows show how their potential interconnectivity and discussion in each category is provided as follows.

### 5.1. Standardization

#### 5.1.1. Unification

Despite numerous auditing solutions, most assessment tools aim to identify a specific firmware vulnerability. In this context, auditing frameworks must be unified to provide a comprehensive vulnerability evaluation instead of developing isolated tools and firmware-specific solutions [100,144]. The development of self-evolving, extendable, platform-independent automated mechanisms will further facilitate firmware auditing, testing and validation for the IoT community. Modular unified frameworks, that can incorporate static and dynamic ensembles, may lead to the implementation of hybrid approaches and offer better scalability and efficiency [48,125]. Developed frameworks should broadly cover the auditing of hardware, firmware, and connectivity aspects of IoT devices for regulatory compliance, configuration, and seamless deployment.

#### 5.1.2. Firmware Stack and Instruction Set

Firmware stacks and instruction set analysis could also benefit from unification and greater standardization. Currently there is no unified IoT firmware architecture [129] and, although there are commercial reasons behind their inherent variability, having a unified architecture would make security analysis significantly easier. Unification also requires translation, interpretation, and mapping to associate any abstract (IoT-specific) commands to multiple underlying architectures allowing greater automation [145]. Firmware vendors must also agree to a unified machine-independent stack for firmware development in order to have greater standardization. The impact of these measures must be carefully weighed; while standardization endeavors may not result in the development of new firmware stacks, the ability to fully support existing supported stacks such as the Unified Extensible Firmware Interface (UEFI) would be helpful [146,147]. To conclude, the design of a firmware stack that can be used as a model for IoT-ware and future applications would immensely benefit practitioners in industry and academia.

### 5.2. Technical Redressal

#### 5.2.1. Analysis Methodologies

As previously established, reverse engineering requires further research since certain processor architectures do not have any associated de-compilers and cannot be analyzed. Incorporating ML as part of reverse engineering will provide automation of the tasks by connecting relevant pieces of information for human analysis. Specifically, ML can automate training by using identified matching problems in several architectures and aggregated learning models through federated learning (FL). FL can be employed to fuse the extracted ML models at an aggregation server and expedite reverse engineering tasks of separate vulnerability classes, holistically identifying threats across the entire set of features offered by the device type [148].

As mentioned in the earlier discussion, dynamic analysis requires emulation across multiple architectures, which is also far from flawless and may crash due to the unavailability of NVRAM parameters [129]. Extending QEMU and similar emulator technologies, re-enforced by ML, can help identify any existing vulnerability patterns. Emulation can also leverage blockchain assisted federated learning to incentivize local model training and regularly update global vulnerability classification models [57]. Crafting statistical input features in traditional ML systems can again be manually intensive; the evolving narratives have therefore resorted to deep learning (DL) structures as a viable alternative. Firmware security analysis can also leverage DL techniques to feed raw data comprising device properties such as domain of use, instruction-set, firmware architecture, DMA specification, peripheral device composition, and vendor-specific information for automatic retrieval of usable features to support classifier training and vulnerability identification. Some studies have already shown promising results in the application of DL approaches for static binary as well as dynamic analysis to inspect vulnerability type signatures and similarities [149,150]. With a handful of basic studies, DL incorporation in firmware vulnerability assessment is still nascent and open for further academic and industrial investigation. Sufficient training data for ML and DL structures would, however, require greater data sharing between vendors, auditors and regulatory bodies.

#### 5.2.2. Secure Ecosystem

Prominent IoT security organizations such as the European Union Agency for Cybersecurity (ENISA), OWASP, IoTSF, and Symantec recommend IoT firmware updating as one of the most important steps towards improving IoT security. Several IoT secure update protocols, including the IETF SUIT [134] standard, have been suggested by prior studies [16,34,89,102,108]. A standardized firmware update framework for this purpose can protect against one of the biggest attack vectors in the IoT paradigm [137]. Blockchain technology can also be used to store authentic copies of firmware made available to participating nodes and customers for over-the-air retrieval [63]. As previously discussed, blockchain technology using several different publishing, incentive, and peer to peer models has been used in firmware storage and delivery [57,58,59,102]. Incorporating blockchain transactions during firmware authentication and download can also aid accountability and reduce instances of compromised image updates being downloaded by everyday users, while firmware update distribution incentives can also reward participating vendor blockchain peers. Verification of downloaded images and general update delivery mechanism are only a few of the suggested efforts in existing research [62,63,64,101] with significant work required to translate generic blockchain assisted models in real-world firmware security scenarios. Blockchain structures can also be used as a knowledgebase repository of vulnerability signatures, storage of locally generated ML/DL classifiers, publication of unknown vulnerability information and integration with regulatory oversight bodies to increase consumer confidence in IoT-ware.

#### 5.2.3. Tool Management and Data Collection

The typical performance expectations from the previously considered vulnerability analysis tools are not on par with evolving vulnerabilities and are affected by firmware unpacking or availability issues. In this context, tool compatibility with IoT-ware is important as some were not built for embedded or IoT devices [30]. Furthermore, limited or altogether absent support and guidance remain a constant concern. A few of the analysis platforms mentioned, such as Firmalice, have had no compatibility studies associated with them, while others such as IDA Pro, are either costly or proprietary. Tools may also report a high false positive/negative rate due to the versatility of IoT devices [93]. Additionally, much of the work performed in firmware analysis has typically focused on Linux-based systems, which are popular due to availability of open source and free tools [116], making the investigation of Windows-based and other platforms quite complex. A growing proportion of firmware is, however, based on various operating systems, therefore future research must propose novel methodologies for finding and resolving the respective vulnerabilities, regardless of OS platform. Essentially, understanding the behavior of the device architecture, unpacking and format analysis, and finally understanding the code behavior can jointly improve vulnerability auditing. This is an ongoing challenge due to limited success of current standardization efforts and the heavy reliance on the developer and vendor priorities.

Aggregated efforts are also required towards firmware metadata collection, which is an essential factor in reverse engineering for firmware analysis and towards reducing the overall analysis time by recording and referring to typical vulnerabilities. Blockchain is a promising technology in this regard; with its immutability, verifiability, and storage features, metadata can be recorded on public and private blockchains and made accessible to the vulnerability research community for reference. A more progressive approach could, therefore, be to use blockchain-based repository, offering greater unification of resources rather than the traditional vendor provided online datasheet. In essence, there is a substantive need for a publicly available firmware database, accompanied by the metadata for the respective releases, to allow researchers and security experts to benefit from it as well as share their expertise to benefit vendors.

### 5.3. Design Innovations

#### 5.3.1. Operating Systems and Platforms

OS compatibility with the underlying hardware architecture is important from a security perspective [2]. Any removal of libraries and packages that are unnecessary for device operation reduces the possibility of potential exploits. While there have been concerns by developers against integration of standardized platforms such as UEFI in IoT-ware, the domain is still open for further investigation [151]. Employing UEFI in IoT for firmware development would allow developers to reduce the time to market, given the usage of UEFI is well-known in traditional computing devices, since rapid product development cycles have had an impact on the ability of OS designers to economically realize IoT-specific operating system. As a result, current IoT devices mostly use stripped down versions of existing OSes primarily designed for other purposes/systems while only few developments such as Contiki, Android things, or LiteOS offer IoT-specific systems.

#### 5.3.2. Emulation Support

The current IoT ecosystem relies significantly on security through secrecy. While this may be acting as a deterrent for some attacks, making the firmware of IoT devices public would bring in the support of the research community and facilitate emulation designing and long-term support [38,98]. Providing the source code and design of the firmware will encourage contribution to standardize and broaden the scope of emulator testing. Code testing, which is a vital part of the development process, focuses typically on ensuring the delivery of core logic rather than security provision. Static analysis can also be used during the development phase while dynamic analysis techniques after the product is developed for validation and testing purposes [31,66]. Vendor-emulation software used for IoT-ware testing and quality control can expedite vulnerability analysis and reduce time gap in patching zero-day vulnerabilities if made available to regulatory bodies open-source consortiums.

The role of emerging technologies such as machine learning and blockchain in addressing the technical challenges is highlighted in Table 7.

Apart from the research avenues and open challenges discussed, it is important to acknowledge the hardware, software, and space constraints encompassing IoT-ware. Limited resources can greatly influence the applicability of vulnerability identification, assessment, and mitigation measures as highlighted in Section 3.9. A concerted effort by stakeholders, including vendors, manufacturers, developers, and testers, can aid security improvements by allowing embedding security vulnerability analysis as a crucial aspect of product development.

## 6. Conclusions

The prevalent use of IoT devices to simplify everyday life to achieve automation is increasingly apparent. However, safe everyday device operation requires an adequate level of security. Improving IoT firmware security can provide much-needed assurance to IoT users against security threats. The present work discusses concepts that emphasize the importance of identifying, analyzing, and mitigating security threats specific to IoT firmware. This work explores fundamentals in firmware vulnerability identification, exploring static, dynamic and hybrid auditing techniques as well as state-of-the-art solutions available to counter the insecurity of IoT devices. In parallel, we present a discussion of open challenges and propose recommendations influenced by contemporary technologies, including machine learning, deep learning, federated platforms, and blockchain technology, to give an overall view of IoT-ware vulnerabilities. The paper also acknowledges the need for greater resource unification, standardization, and regulatory guidance from IoT vendors, developers, integrators, and other stakeholders to adequately address current and future security concerns.

## Figures and Tables

**Table 1:** Related contributions in IoT firmware security *.

Domain	Incorporation				References
	Monolithic	Cross-domain	Standards and reg.	Security survey	
Interface security	✓	✓	✓	ƥ	[33,34,35,36]
Firmware auditing	✓	ƥ	ƥ	ƥ	[17,24,29,37,38,39,40,41]
Reverse engineering	✓	✓	X	ƥ	[33,36]
Threat analysis	✓	✓	ƥ	✓	[36,42,43,44]
Tools and testbeds	ƥ	ƥ	✓	ƥ	[2,4,44,45,46,47,48,49,50,51,52,53,54,55,56]
Distributed ledgers	✓	✓	✓	✓	[45,57,58,59,60,61,62,63,64,65,66,67,68]
Machine learning	ƥ	✓	X	X	[31,45,69,70,71,72]
Commercial developments	✓	ƥ	✓	ƥ	[73,74,75]
Remote attestation	✓	X	X	ƥ	[42,76,77,78,79]

* Related work: ✓ Comprehensive studies, ƥ Partial work supporting primary avenue, X Non-existent/non-applicable.

**Table 2:** Firmware reverse engineering—prominent tools and techniques *.

Tool	Operational Domain	Features	Limitations
Binwalk [116]	FU	Firmware analysis, extraction, file system identification, entropy comparison	Limited firmware extraction, recursive unpacking
BANG [117]	FU	Recursive unpacking for approximately 130 file types	Inconsistent support
FMK [118]	FU	Firmware unpacking and extraction and repacking specific to Linux.	Insufficient support, supports only Linux platforms
FACT [119]	MRE, FU	Automatic, extensible basic firmware analysis and comparison to	Limited to static analysis, and to certain Linux distributions, resource heavy
ANGR [120]	MRE, FU	Framework for binary analysis using CFG	Complex usability, limited Windows binary support
Binary Ninja [121]	MRE, FD	Binary analysis with intermediate language supporting multiple platforms, with GUI	Closed source. Limited support for dynamic analysis
Radare2 [122]	FD	Binary analysis, disassembling and debugging	Difficult to learn, and analyze complex code
Ghidra [123]	FD	Open-source analysis and de-compilation tool, supporting multiple platforms	Supports limited architectures and de-buggers, slow performance
KLEE [48]	MRE, FD	Symbolic VM based on LLVM compiler support	Resource heavy
FAT [49]	MRE	Built atop multiple analysis and reverse engineering tools	Compatibility issues of base-tools with Linux
IDA Pro [124]	MRE	Powerful interactive disassembler, debugger, support for multiple architectures	High cost, closed source, basic GUI
QEMU [125]	MRE	Efficient open-source emulator, and virtualization for Linux platforms	Limited GUI, only Linux support
AFL [126]	MRE	Security oriented brute-force fuzzer employing generic algorithms	Requires target input to learn and improve

* MRE: Multiple Reverse engineering Tasks, FA: Firmware Acquisition, FU: Firmware Unpacking, FD: Firmware Decompiling.

**Table 3:** Firmware auditing schemes.

Auditing Feature	Static	Dynamic
Methodology	Code scanning (manual, semi-automated)	Execution-based behavior analysis
De-compilation	Limited applicability as de-compiler may not be available or produce false output	No requirement for code de-compilation
False Positives/Errors	High rate of false positives	N/A
Firmware Acquisition	Acquiring device firmware is necessary	There is no need to acquire firmware if the device is locally or remotely available
Manifest	Desired but not necessary	Necessary for virtualization
Non-exploitable code	Non-exploitable code cannot be analyzed	Cannot find unexploited code
Physical device access	Physical access to devices is not needed	IoT device or firmware emulation required
Run-time Insights	No real-time code execution information; problems due to run-time vulnerabilities.	Can provide additional insights on input data/execution during run-time
Scalability	Possible to automate if a large repository of device firmware is available	Can be achieved with greater virtualization
Unused Code	Unused code can be inspected	Not feasible to identify vulnerabilities in unused code of program
Virtualization	Virtualization is not needed	Virtualization needed for manifest/meta-data
Vulnerability Focus	Buffer overflows, memory corruption, segmentation errors, uninitialized variables	Any type of vulnerability can be inspected by running relevant code

**Table 4:** Auditing strategies and tools for static analysis *.

Tool	Year	Analysis Method	Target Vulnerability	SC.	UV.	PV.	Architecture
Woodpecker [135]	2012	Code analysis	Permission leaks	N/A	N/A	Yes	Android
Correlation engine [81]	2014	Vulnerability correlations	Any	Yes	Yes	Yes	Multiple
Firmalice [108]	2015	Symbolic execution	Authentication	Yes	Yes	No	Multiple
PIE [69]	2015	Parsing identification	Bugs, protocol specs, commands	No	Yes	No	Multiple
ANGR [120]	2016	Binary control flow graphs	Any	Yes	Partly	Yes	Limited
Genius [136]	2016	Control flow graphs	Any	Yes	Yes	No	Multiple
Gemini [40]	2017	Neural network	Any	Yes	Partly	Yes	Multiple
HumIDIfy [45]	2017	Machine learning	Finding hidden functionality	No	Partly	Yes	Multiple
Stringer [134]	2017	Automated analysis	Finding backdoors	Yes	No	No	Multiple
FirmUp [82]	2018	Program slicing	Multi-domain	Yes	Yes	Partly	Multiple
UFO [137]	2018	Shell script dependency	Multi-domain	Yes	Yes	Yes	Multiple
Two-stager [41]	2019	Code similarity	Any	Yes	Yes	No	Multiple

* SC: Scalability issue, UV: Unknown Vulnerability Detection, PV: Platform Versatility.

**Table 6:** Vulnerability coverage—existing techniques.

Vulnerab. Influence	Exploitation Axes	Auditing Technique		Supported Platform				Architecture Compatibility			
		Static	Dynamic	Linux	Windows	RTOS	Other	ARM	MIPS	X86	Other
System Property	Memory corruption	[52,69,82]	[41,51,129,142]	[37,41,51,52,69,82,129,142]	[41,69,82,129,142]	[37,41,56,69,82,129]	[51,69,82,129,142]	[41,51,69,82,129,142]	[53,69,82,129,142]	[14,41,83,86,95]	[82,129,142]
	Taint vulnerability	[52,53]	-	[52,53]	[53]	[53]	[53]	[52,53]	[52,53]	[53]	[53]
Access Mechanisms	Authentic./Backdoor	[39,45,81,108,122,137]	[99]	[45,81,99,108,137]	[108]	[108]	[108]	[45,81,99,108,137]	[45,81,99,108,137]	[99,108]	[99,108]
	Weak password	-	[45,81,122,137]	-	[81]	-	[45,81]				
Comp. Re-use	Obsolete components	[54,55]	[104]	[50,54,55,104]	[104]	[104]	[104]	[50,54,55,104]	[50,54,55,104]	[50,54,55,104]	[50,54,55,104]
Network Interface	Insecure interfaces	[137]	[69]	[69,137]	-	-	[69]	[69,137]	[69,137]	[69,137]	[69,137]
	Weak firewall	[45]	-	[45]	-	-	-	[45]	[45]	-	-
Image Mgmt.	Web Services, Storage	[81]	[81]	[81]	-	[81]	-	[81]	[81]	[81]	[81]
Regulatory Compliance, User awareness	Hard-coded credentials	[39,45,56,81,143]	-	[45,56,81]	-	-	-	[45,81]	[45,81]	[81]	[81]
	Information leakage	-	[99]	[99]	-	-	-	[99]	[99]	[99]	[99]
Adversary Vectors	Side-channeling	[7,33,38,85]	[142]	[3,38,85,94]	[108]	[108]	[108]	[3,7,38,94]	[3,7,38,94]	[99,108]	[99,108]

RTOS: Real-Time Operating System; ARM: Advanced RISC Machine; MIPS: Microprocessor without Interlocked Pipeline Stages; x86: processor architecture, developed by Intel and AMD.

**Table 7:** The overview of application areas for blockchain and machine learning in IoT firmware security.

Technology	Application Area	Adaptations and Recommendations
ML and DL	Automated reverse engineering [148]	Reverse engineering tasks require greater automation using intelligent code analysis and ML/federated learning models.
ML and DL	Improved emulation [31,57,66]	Improvement in emulation systems can help to extend dynamic analysis to architectures where static analysis is the only primitive available. Self-evolving emulators based on prediction DL models with automated selection of architecture-dependent parameters can also be helpful.
ML and DL	Identifying vulnerability patterns [149,150] Auditing framework [57]	ML and DL technologies can aid in pattern/signature recognition while federated learning platforms can ensure low-latency local model generation for global classifiers. FL can ensure greater data privacy and anonymity while the framework can utilize blockchain peers for verifiability and immutability.
Blockchain	Secure firmware update [62,63,64,101]	OTA updates need to have sufficient security guarantees; blockchain technology can be employed as a promising alternative to deliver OTA updates.
Blockchain	Image storage and verification [57,58,59,102]	Blockchain technology can be used for storage and delivery of firmware images offering verifiability and accountability for regulatory bodies.
Blockchain	Metadata collection	Metadata and manifest information should be available in a central repository and verifiable for authenticity. Blockchain can be leveraged for this purpose.

## Data Availability

Data are contained within the article.
